# Therapeutic utility of engineered myeloid cells in the tumor microenvironment

**DOI:** 10.1038/s41417-023-00600-7

**Published:** 2023-02-28

**Authors:** Alessandro Canella, Prajwal Rajappa

**Affiliations:** 1 Institute for Genomic Medicine, Nationwide Children’s Hospital, Columbus, OH USA; 2 Department of Pediatrics and Neurological Surgery, The Ohio State University Wexner Medical Center, Columbus, OH USA

**Keywords:** Immunization, Tumour immunology, Cancer microenvironment

## Abstract

Despite promising results shown in hematologic tumors, immunotherapies for the treatment of solid tumors have mostly failed so far. The immunosuppressive tumor microenvironment and phenotype of tumor infiltrating macrophages are among the more prevalent reasons for this failure. Tumor associated macrophages (TAMs, M2-macrophages) are circulating myeloid cells recruited to the local tumor microenvironment, and together with regulatory T cells (T-regs), are reprogrammed to become immune suppressive. This results in the inactivation or hampered recruitment of cytotoxic CD8 + T and Natural Killer (NK) cells. Recently, attempts have been made to try to leverage specific myeloid functions and properties, including their ability to reach the TME and to mediate the phagocytosis of cancer cells. Additionally, myeloid cells have been used for drug delivery and reprogramming the tumor microenvironment in cancer patients. This approach, together with the advancements in genome editing, paved the way for the development of novel cell-mediated immunotherapies. This article focuses on the latest studies that detail the therapeutic properties of genetically engineered or pharmacologically modulated myeloid cells in cancer preclinical models, limitations, pitfalls, and evaluations of these approaches in patients with cancer.

## Introduction

The natural immunosurveillance system is composed of two parts: the adaptive and the innate immune systems. B and T lymphocytes are adaptive immune cells, while natural killer (NKs), dendritic cells (DCs), and myeloid cells are innate immune cells. B cells are responsible for the humoral response against cancer cells, while T, NK, DC, and myeloid cells induce a cell-mediated immune response.

Cellular immunotherapies or “adoptive cell therapies” are among the most advanced treatments that have been developed so far for cancer patients. Of note, autologous immune cells are isolated from patients and reinfused to improve anti-cancer specificity and efficacy. The first cellular immunotherapies were developed in 1993 and focused on engineering T-cells with a chimeric antigen receptor T (CAR-T) [1]. In 2012, the first patient was treated with CAR-T cells and, in 2014, CAR-T received the “breakthrough drug designation” status by the FDA for the treatment of relapsed/refractory B-cell lymphoblastic leukemia. Currently, the FDA has approved CAR-T therapies for the treatment of a multitude of hematological malignancies, mostly lymphomas, but also multiple myeloma and acute lymphoblastic leukemia (ALL). More recently in 2021, the FDA granted the “breakthrough drug designation” for the use of tumor infiltrating lymphocytes (TILs) for the treatment of cervical cancer [2]. Overall, the initial pre-clinical evaluation of T cell immunotherapies had shown positive and encouraging results and was on track to quickly revolutionize the treatment of hematological tumors. On the contrary, several clinical trials have exposed the limited efficacy of T cells immunotherapies in solid tumors. CAR-T therapies demonstrated limited long-term efficacy, development of severe side effects, difficulties in penetrating the immunosuppressive tumor microenvironment (TME), and the onset of mutations in cancer cells during tumor progression potentiated resistance to the therapy (antigen escape) [3–5].

Despite these limitations, data collected in pre-clinical and clinical evaluations on CAR-T therapies encouraged the scientific community to develop and test various adaptive and innate cell-based immunotherapies. Among them, engineered myeloid cells together with NK cells [6] are some of the most novel therapies in the field.

Historically, myeloid cells were considered targets for the therapy of tumors with an immunosuppressive TME [7–10], but they offered several advantages in the utility of cellular-immunotherapy. They can be delivered systemically and efficiently recruited to the TME [11], where they home and demonstrate stability for weeks and are important players in the crosstalk between innate and adaptive immunity [12].

**Fig. 1:**
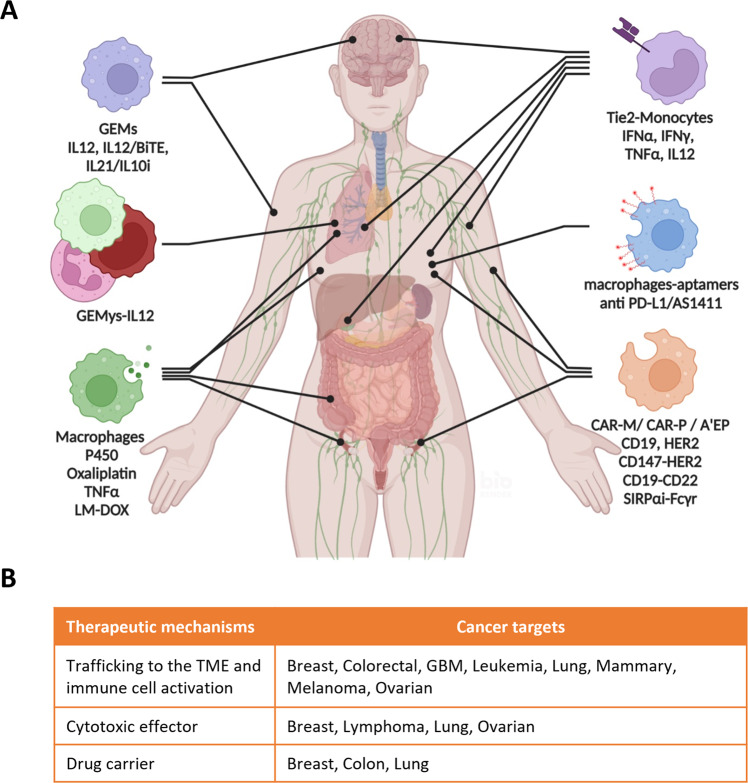
Therapeutic use of engineered myeloid cells in cancer. **A** Summary of the most relevant pre-clinical evaluations of the treatment of tumors with engineered myeloid cells. Illustration created with BioRender.com. **B** Therapeutic mechanisms of engineered myeloid cells.

Here, we trace the evolution of cell-mediated immunotherapies based on genetically modified myeloid cells from the preclinical investigations (Fig.1, Table 1) to clinical trials (Table 2). Particularly, we dissect the technical aspects – as well as the results and pitfalls of this approach – in the most relevant scientific literature in the field.

**Table 1:** Summary of the most relevant pre-clinical evaluations of genetically modified myeloid cells in cancer therapy.

Target	Mechanism	Engineered cells	Gene	Method	Reference
Breast	Immune cell activation	Tie2-monocytes	IFNα	LV^i^	Escobar et al. [27]
	Immune cell activation	Macrophages (CAR-M)	HER2	LV	Zhang et al. [58]
	Cytotoxic effector	Macrophages	PD-L1, AS1411	MGB^ii^	Qian et al. [65]
	Drug carrier	Macrophages	Oxaliplatin	CI^iii^	Huang Y. et al. [72]
Colon	Drug carrier	Macrophages	TNFα	LV	Huang L. et al. [73]
Colorectal	Immune cell activation	Tie2-monocytes	IFNα	LV	Catarinella et al. [28]
GBM	Immune cell activation	Tie2-monocytes	IFNα	LV	De Palma et al. [26]
	Immune cell activation	Tie2-monocytes	IFNα, IL12	LV	Birocchi et al. [33]
	Trafficking to the TME	Macrophages	IL10i, IL21	LV	Moyes et al. [41]
	Immune cell activation	Macrophages	IL12	LV	Brempelis et al. [42]
	Immune cell activation	Macrophages	BiTE, IL12	LV	Gardell et al. [43]
Leukemia	Immune cell activation	Tie2-monocytes	IFNα	LV	Escobar et al. [34]
	Immune cell activation	Tie2-monocytes	IFNγ, TNFα	LV	Mucci et al. [35]
Lymphoma	Cytotoxic effector	Macrophages (CAR-P)	CD19, CD22	LV	Morissey et al. [57]
Lung	Immune cell activation	Myeloid cells	IL12	LV	Kaczanowska et al. [44]
	Cytotoxic effector	Macrophages (A’EP)	SIRPαi, Fcy-r	LV,Ab^iv^	Alvey et al. [68]
	Drug carrier	Macrophages	LM-DOX	LIP^v^	Guo et al. [75]
Mammary	Immune cell activation	Tie2-monocytes	IFNα	LV	De Palma et al. [26]
Melanoma	Immune cell activation	Macrophages	IL12	LV	Brempelis et al. [42]
Ovarian	Cytotoxic effector	Macrophages	Cyt. P450	AV^vi^	Kan et al. [45]
	Immune cell activation	Macrophages (CAR-M)	HER2	LV	Klichinsky et al. [59]

^i^Lentivirus, ^ii^Metabolic Glycan Biosynthesis, ^iii^co-incubation, ^iv^antibodies, ^v^anchoring lipopolysaccharides by coincubation, ^vi^adenovirus.

**Table 2:** Clinical trials with genetically modified myeloid cells in cancer.

Clinical trial n.	time	phase	Engineered cells	Tumor
NCT03875495	2019–2022	I/IIa	Tie2-macrophages	Multiple myeloma
NCT03866109	2019–2022	I/IIa	Tie2-macrophages	GBM [32]
NCT04660929	2021–2023	I	CAR-M	29 different tumors
NCT05007379	2021–2023		CAR-M	Breast cancer

Source: *clinicaltrials.gov*.

## Genetically engineered myeloid cells in cancer therapy

The first relevant investigation that paved the way for the therapeutic use of genetically engineered myeloid cells in vivo was published in 2001 by Wu and colleagues [13]. In this study, the researchers demonstrated that airway engraftment of engineered macrophages for the release of Interferon-γ (IFNγ) was able to reactivate the immune response in the lungs of immune compromised mice (scid). The engineered cells trafficked to the lungs and efficiently released IFNγ, which enhanced the production of other pro-inflammatory cytokines and potentiated the immune stimulation of phagocytosis for up to 14 days. Subsequent studies utilizing the transplantation of autologous hematopoietic progenitors (HPCs) in patients on high dose chemotherapy has been used as a well-tolerated therapeutic strategy for the treatment of solid (breast, ovarian) [14–17] or hematological malignancies [18, 19]. Moreover, the advances in viral strategies to regulate gene expression in hematopoietic cells [20–23] boosted the use of engineered myeloid cells in therapy, making them a promising novel immunotherapeutic field to explore, especially for the treatment of malignancies underscored by immunosuppressive tumor microenvironments (TME) [24]. More recently, myeloid cells have been also genetically modified to express fluorescent proteins, allowing in vivo investigation of the maturation, distribution, trafficking, and recruitment to the TME during tumor progression [11, 25].

### Tie2-monocytes engineered to release pro-inflammatory cytokines

A landmark study, published in 2008, proved for the first time the potential in vivo application of engineered monocytes in cancer therapy for the treatment of glioma and mammary tumors [26]. The pre-clinical strategy was based on the natural peritumoral recruitment of a subpopulation of Tie2-monocytes, expressing the angiopoietin receptor, recruited to the TME by angiogenic-related hypoxic stimuli. By infection with lentiviral particles, the investigators induced the overexpression of interferon alpha (IFNα), whose regulation was mediated by the enhancer/promoter of Tie2, into hematopoietic progenitors (HPCs) isolated from murine bone marrow. The engineered hematopoietic progenitors, systemically engrafted in athymic mice, differentiated into Tie2-monocytes-IFNα. Eight weeks after the HPCs transplantation, mice were engrafted with U87 cancer cells, and the tumor progression was evaluated. The treatment induced a remarkable pro-apoptotic and anti-angiogenic effect on cancer cells without showing any toxicity in rodents. Furthermore, the effect in vivo of genetically modified monocytes for the release of IFNα into a mammary immunocompetent cancer model demonstrated increased infiltration of myeloid CD4 + and CD8 + T cells in the tumor microenvironment (TME) and decreased tumor burden [26]. This was the first time that the biological effect of genetically engineered myeloid cells on the adaptive immune system was reported in the scientific literature. In this study, they also demonstrated that the infection of syngeneic HPCs with lentiviral particles and the intravenous delivery into breast cancer immunocompetent murine models induced the accumulation of Tie2-monocytes-IFNα. This was associated with impaired tumor progression and the reduction of lung metastases. Of relevance, results in vivo showed immune cell activation of CD4 + and CD8 + T cells in association with the animals treated with autologous and engineered HPCs [27]. The same therapeutic concept (Tie2-monocytes releasing IFNα) was also evaluated in vivo in a colorectal cancer model [28]. In this study, cancer cells were intrasplenically injected in immunocompetent mice (CB6 strain) 8 days before the systemic delivery (via iv injection) of engineered HPCs. Similarly, this study showed impaired tumor progression and the intratumoral activation of IFNα inducible genes with a significant survival advantage. In addition, the treatment did not induce toxicities in treated animals and the authors did not detect any accumulation of genetically modified cells in other organs; thus, the safety of the treatment in rodents was confirmed.

Studies have also demonstrated the challenges associated with treating central nervous system (CNS) tumors due to the presence of the blood brain barrier (BBB) that affects drug delivery and the immunosuppressive environment, which supports tumor progression by potentiating drug resistance mechanisms of tumor escape [29]. In addition, the novel immunotherapies based on the delivery of pro-inflammatory cytokines face several crucial challenges when used for the treatment of CNS tumors such as the need to restrict the biological effect on cancer cells to reduce the toxicity and to limit off-target effects in patients [30, 31]. Therefore, major efforts have been recently devoted to the design of treatments where the release of cytokines can be inducible or fine-tuned during the treatment course.

A phase I/IIa clinical trial was recently designed to evaluate the toxicity of Temferon (macrophages-IFNα + /Tie2 + ) in patients newly diagnosed for glioblastoma multiforme (GBM). The trial confirmed what was reported previously in vivo. The treatment was well tolerated and the engineered myeloid cells were detected in the bone marrow and peripheral blood up to 14 days post engraftment [32]. In addition, the team that developed the Tie2-monocyte model for cancer therapy [26–28, 32], recently tested the effect of myeloid cells engineered for the inducible release of IFNα and Interleukin-12 (IL12) in glioblastoma (GBM) [33]. In summary, they generated gene fusions of IFNα and IL12 with destabilizing domains (DDs). The DD-fusion generates unstable and unfolded proteins which are degraded by proteasomes, but the addition of DD-binging molecules stabilizes the fusion protein and avoids proteasomal degradation. Therefore, this allows for the secretion of active pro-inflammatory cytokines. The main purpose of this strategy was to minimize the toxicity by controlling the release of pro-inflammatory cytokines and minimize their cellular activity in off-target organs. In vivo, they verified the therapeutic efficacy of the inducible treatment in combination with TMP (trimethoprim, DDs stabilizer agent), which was able to block the tumor progression and reprogram immunosuppressive TAMs to a pro-immune activation state and inhibit the expression of genes associated with T cell exhaustion. Interestingly, they also proved the flexibility of their therapeutic approach by showing the inhibition of the tumor progression in GBM by inducible Tie2-monocytes-IL2 [33].

Intriguingly, reprogramming immune cells using Tie2-monocytes-IFNα was also investigated for the treatment of hematological malignancies associated with the development of an immunosuppressive phenotype during tumor progression. Escobar et al. investigated the therapeutic effect of the engineered monocytes in B cell acute lymphoblastic leukemia (B-ALL) as a single agent, and in combination with CTLA4 inhibition, or with CAR-T cells. The combination of the two therapies showed improved survival and activation of cytotoxic T cells response in semi-immunocompromised mice (C57Bl/6 Ly45.1/Ly45.2). To demonstrate therapeutic effect, the IFNα monocytes were implanted in semi-immunocompromised animals, but 6 weeks prior to tumor implantation. In addition, the therapy demonstrated the best efficacy in vivo when tested in combination with the immunocheckpoint CTLA4 blockade [34]. To assess the safety of Tie-2 HPCs-IFNα treatment in patients affected by hematological malignancies, a phase I/IIa clinical trial was conducted in multiple myeloma (NCT03875495), but the results are not available yet (Table 2). To further improve the therapeutic efficacy of the strategy used in B-ALL, the preclinical use of Tie2-monocytes for the co-release of interferon gamma (IFNγ) and tumor necrosis factor alpha (TNFα) was tested in vivo [35]. Immunocompetent mice were engrafted with B-ALL cells, treated with vincristine, sub-lethally irradiated, and infused with engineered monocytes. The treatment of leukemia mice with Tie2-monocytes, engineered for the release of IFNγ and TNFα, induced the activation of the CD8 + effector T cells, but also of MHC II + memory T cells and significantly delayed tumor progression. However, most of the effect in vivo seemed to be attributed to the treatment with IFNγ cells. Furthermore, the over stimulation with IFNγ ultimately led to the downregulation of IFNγ receptors, CD8 + T cell downregulation, and tumor relapse [35].

### Genetically engineered macrophages (GEMs) and bone marrow-derived myeloid cells (GEMys) to release pro-inflammatory cytokines

Interleukin-12 is vital for the crosstalk between innate and adaptive immunity and is a potent enhancer of the anti-tumor immunity in vivo. Moreover, IL12 is an effective stimulator of the activation of cytotoxic T and NK cells. In cytotoxic immune cells, IL12 also promotes proliferation and release of IFNγ. In tumors with immunosuppressive TMEs, interferon-γ is a positive regulator of the activation of dendritic cells (DCs), NK and cytotoxic T cells, and pro-immunostimulatory macrophages. On the counterpart, IFNγ is a negative regulator of activation of immunosuppressive regulatory T lymphocytes and T helper 17 cells (Th17) [36, 37]. In cancer patients, the treatment with recombinant IL12 has been demonstrated to be effective, but it has also been associated with toxicity [38, 39]. Therefore, the challenge for IL12 and IFNγ immunotherapies for the treatment of cancer is to be able to trigger a strong recruitment and activation of anti-cancer adaptive immunity within the TME while minimizing the toxicity. In patients with recurrent high-grade glioma (rHGG) enrolled in a phase I clinical trial (NCT02026271) for the delivery of IL12 with replication-incompetent adenovirus (Ad–RTS–hIL-12), results demonstrated safety and better tolerance to treatment with increased levels of IL12 and local release of IFNγ and increased infiltration of CD8 + T cells [40].

Recent innovative studies have suggested for the first time, the use of genetically engineered macrophages (GEMs) for the release of cytokines in the GBM microenvironment as an alternative to the use of adenovirus for the release of IL12 in cancer treatment [41]. In a pioneer study designed as a proof of concept using engineered macrophages in GBM therapy, the cells were engineered to silence IL-10 and PD-L1 while overexpressing sTbRII and IL-21. In an intracranial stereotactic GBM model (U87 in NSG immunocompromised mice), GEMs delivered by intratumoral injection have been showed to home to the TME. In a recent study, primary derived macrophages were genetically modified with lentivirus particles for the overexpression and release of IL12, and then reinfused in syngeneic immunocompetent and tumor bearing mice for the treatment of GBM and Melanoma [42]. The local delivery by intratumoral injections of GEMs in GBM mouse models, either intracranial or subcutaneous, proved homing of the engineered cells up to 20 days post-injection to the TME. The study also demonstrated the TME recruitment of systemically delivered GEMys tumor progression arrest in a subcutaneous melanoma model. Treatments with GEMs were well tolerated, and the beneficial effect of the recruitment of cytotoxic T and NK cells to the tumor site was also reported in RCAS/TVA glioma model. However, no data on the overall survival of the animals was reported [42]. To fill the gap in knowledge on human cells, a follow up study, from the same team, proved the ability of human GEMs-IL12/BiTE to activate human T cells and better recognize cancer cells. The myeloid cells were engineered for the release of IL12 and Bispecific T cells engagers (BiTEs). The role of BiTEs is to facilitate the interaction between cytotoxic T cells and cancer cells. Interestingly, the intratumor injection of human GEMs-IL12/BiTE, followed by the systemic injection (iv) of activated human T cells in EGFRvIII-U87 subcutaneous NSG murine model, demonstrated increased killing of cancer cells and a reduction of the tumor progression, compared to GBM animals treated with systemic injection of activated human T cells only [43]. Overall, pre-clinical data generated on this novel therapeutic concept were encouraging and warranted further investigation of GEM-based therapies. However, the fact that the best survival results were demonstrated when the cells were delivered by intratumoral injection and in immunocompromised mouse models raised a red flag on the translational relevance of the treatment. Particularly, the treatment is questionable for cancers like the CNS tumors, where treatment by intratumoral injection is more challenging.

A very elegant work on the pre-clinical use of novel myeloid-mediated immunotherapies for the release of IL12 to the TME was published by Sabina Kaczanowska et. al. in 2021 [44]. The project was focused on the pre-clinical evaluation of the therapeutic efficacy of primary bone marrow-derived myeloid cells genetically engineered for the release of IL12 (GEMys-IL12) in vivo in a pre-metastatic and immunocompetent lung cancer murine model. The study pointed out the presence of an immunosuppressive tumor microenvironment in lung cancer to restrict cytotoxic T and NK cells recruitment and anti-cancer cells activity. Additionally, the study revealed the immunosuppression was essentially regulated by M2-macrophages. The systemic engraftment of GEMys-IL12 in lung cancer mice proved their recruitment to the TME, followed by the increased trafficking and activation of T, NK, and dendritic cells, thus influencing the composition and transcriptome of the tumor infiltrated immune cells. Remarkably, the treatment in vivo with GEMys-IL12 was able to block tumor growth, arrest the spreading of metastasis, and improve the overall survival [44]. The engineered myeloid cells were largely composed of monocytes, macrophages, conventional dendritic cells (DCs), and granulocytes. However, the characterization of the myeloid composition of the GEMys was incomplete, with 40–60% of the bone-marrow derived myeloid cells in the samples not clearly defined. In addition, the spike of IL12 and IFNγ after treatment was noted in the lung, but also in the spleen, liver, and peripheral blood. Moreover, the signal dropped significantly at 8–10 days post-treatment, thus suggesting the need for recurrent treatments for a more robust therapeutic effect, even though it might be associated with treatment tolerability or increased toxicity.

Although these innovative cell-mediated immunotherapies have not been tested on patients yet, the encouraging results on the recruitment of myeloid cells at the TME, the low but persistent release of IL12 and IFNγ, and the trafficking and activation of anti-cancer cells in the TME suggest improved tolerability, reduced toxicity, better efficacy of the treatment, and reduced off-target distribution of the therapy. Future therapies based on GEMs and GEMys may replace the use of recombinant cytokines in cancer therapy.

### Myeloid cells engineered to become cytotoxic effectors

Genetically modified myeloid cells were also employed as cytotoxic effectors against cancer cells. In ovarian cancer and in other kinds of solid tumors, the TAMs are known to infiltrate the TME, mostly surrounding the necrotic and hypoxic areas. A different therapeutic strategy, based on human macrophages engineered for the release of human cytochrome P450, was successfully tested for the in vivo treatment of ovarian PDX model in combination with cyclophosphamide (CPA). Briefly, genetically engineered macrophages derived from human monocytes were infected with adenoviral particles for the expression of cytochrome P450 (P450 2B6). Once delivered in ovarian PDX mice, engineered macrophages localized at the TME released P450 to convert CPA into toxic metabolites to induce cancer cell death. This was associated with a 2-fold improved overall survival [45].

Among the numerous cellular-mediated immunotherapies developed and tested in the past, the genetically modified T cells with expression of chimeric antigen receptors, CAR-T cells, were certainly the most promising. One of the most studied modifications has been the expression of the human epidermal growth factor receptor-2, ErbB2 (HER2) in CAR-T cells. HER2 is overexpressed in a host of various tumors: lung, melanoma, gliomas, breast, gastric, and thyroid. In addition, overexpression of HER2 was found in 80 to 100% of cancer tissues from patients, depending on the different kind of tumors investigated [46]. CAR-T was a revolutionary treatment and the first FDA approved gene therapy for the treatment of B cell-lymphomas and acute lymphoblastic leukemia (ALL). So far, this type of immunotherapy has shown prolonged efficacy only in patients affected by a few specific types of lymphomas and leukemias [47]. Several factors responsible for the failure of this therapy have been identified so far. As remarkably reported by Shah and Fry [3], some of the most relevant are the development of resistance mechanisms, limited efficacy, tumor heterogeneity, and secondary mutations evolving during the tumor progression and loss of CAR-T specificity for the target cells. Moreover, isolation of autologous T cells and expansion of post-modification prior to reinfusion in patients can be extremely challenging, and it can ultimately affect the therapeutic efficacy. In addition, the tumor progression in several solid tumors is associated with an accumulation of immunosuppressive T-regs and macrophages which can restrict the recruitment and activation of cytotoxic T cells. These circulating macrophages in the blood stream are recruited to the tumor microenvironment (TME), reprogrammed to become immunosuppressive, and hamper trafficking and activation of cytotoxic T, and natural killer (NK) cells [48–56]. Recently, some studies have combined the advantages of CAR-T therapy and the recruitment of macrophages to the TME to develop an innovative therapeutic strategy where the macrophages were modified to express chimeric receptors, thus becoming CAR-M, or CAR-P. To that end, the first relevant work published on CAR-P was in 2018 [57], where the authors hypothesized that macrophages could be engineered for the expression of chimeric receptors similarly to the T cells and then kill the target cells by phagocytosis (P stands for phagocytes). Macrophages were modified to express a recombinant protein with an extracellular domain, the single-chain antibody variable fragment (scFv), for a specific targeting of CD19 + and CD22 + B cells, and the intracellular domain of the phagocytic receptor Megf10. The engineered myeloid cells were tested in vitro for the killing of Raji cells (human lymphoma cell line), and the co-culture of Raji cells with αCD19 CAR-P cells showed a significant reduction of cancer cells in less than 2 days. This was the first study where engineered myeloid cells were used for the treatment of hematological malignancies, but no experiments in vivo were performed. In addition, those results demonstrated for the first time that the CAR approach could be adopted for other types of immune cells and that CAR-myeloid cells could represent a novel cell-based cancer immunotherapy. Afterwards, the CAR-M therapeutic concept has been evaluated in vivo by Zhang [58] in breast cancer and by Klichinsky [59] in ovarian cancer. In the first paper, HER2-CD147 expression was induced in macrophages, and the engineered cells were reinfused systemically in mice subcutaneously engrafted with breast cancer cells. CD147 is overexpressed in cancer cells, and it is directly involved in different aspects of cancer biology such as tumor progression, metastasis, remodeling of the extracellular matrix in the TME, and gene expression regulation of metalloproteinases (MMPs). HER2-CD147-CAR-M cells demonstrated no cytotoxic activity on cancer cells, but the treatment triggered a cytokine storm in the peripheral blood and in the TME. Overall, the cytokine storm involved the upregulation of pro-inflammatory cytokines IL12 and IFNγ. As a consequence of the reprogrammed immune activation in the TME, the investigators also showed a significant increased trafficking of CD3 + T cells in the TME, a reduction of immunosuppressive myeloid cells, and a reduction of the tumor burden [58]. Mice were treated with 2 injections of CAR-M cells and the results showed robust arrest of the tumor growth for 20 days post treatment, but no results on the survival were reported. In the study, the authors demonstrated the preclinical anti-cancer activity of human CAR-Macrophages (hCAR-Ms) [59]. hCAR-Ms directly killed target cells by phagocytosis and the model was also applied on CD19 + K562 cells using CD3ζ-CAR-Ms and on mesothelin+ and HER2 + K562. This confirms the potential cytotoxic activity on target cancer cells expressing specific markers in solid tumors and hematological malignancies. The activity of human HER2 + CAR-M cells was also tested in vivo in ovarian cancer. NOD-scid mice engrafted with ovarian cancer cells that received one dose of HER2 + CAR-Ms showed significant reduction of the tumor progression, low toxicity, and an improved overall survival. Notably, the investigation by single cell-RNA sequencing of the tumor microenvironment infiltrated by human CAR-Ms in humanized mice demonstrated the reprogramming of the TME to a pro-inflammatory phenotype with the activation and recruitment of activated human T cells and immature dendritic cells [59, 60]. Currently, the activity of CAR-macrophages in patients is under evaluation in the following two clinical trials (Table 2, source *clinicaltrials.org*): a phase I clinical trial evaluating the safety of HER2-CAR-Ms in patients affected by 29 different types of tumors, and a second one aimed to investigate the efficacy of human CAR-M against breast cancer organoids with different expression levels of HER2. No papers have been published so far with the results of the trials.

Myeloid cells have also been chemically modified to enhance the phagocytosis of cancer cells. Aptamers, also defined as “nucleic acid antibodies”, are RNA or single strand DNA that can recognize and bind with high affinity specific targets. They have lower immunogenicity than monoclonal antibodies (mABs), fewer side effects, and they have been already approved by the FDA for targeting VEGF and for the treatment of diseases associated with molecular degeneration (Pegaptanib) [61–63]. The surface modification of macrophages with the addition of nucleic acid aptamers significantly improved the binding and killing of cancer cells by phagocytosis [64]. Moreover, the increased targeting of cancer cells by macrophages chemically modified with aptamers was also associated with an increased expression of MHC class I and II molecules and release of proinflammatory cytokines (TNFα and IL12). The biological effect on cancer cells of macrophages with aptamers was initially evaluated in vitro only, but a study published recently in Molecular Therapy finally addressed the therapeutic effect of anti-cancer macrophages in vivo. The investigators proved the efficacy of treating a breast cancer murine model with lung metastasis with modified macrophages. In the study, RAW264.7 cells were engineered with two different aptamers specific for the binding of PD-L1 (immune-checkpoint) and AS1411 (nucleolin) biomarkers known to be overexpressed on the surface of several kind of cancer cells [65]. The effect in vivo included the increased infiltration of CD8 + and CD4 + T cells, tumor progression arrest, the inhibition of lung metastasis, and a remarkable increased overall survival.

Immunocheckpoint markers are immunoescape mechanisms used by cancer cells to evade cell-mediated killing. Inhibitors of phagocytosis are transmembrane proteins, also defined “don’t eat me” onco-proteins, expressed in cancer cells [66], and CD47 is a “don’t eat me” phagocytosis checkpoint, well expressed in a multitude of cancer types. CD47 binds with high affinity SIRPα molecules expressed on the surface of macrophages to exploit the immunoescape mechanism, contributing to the tumor progression [67]. To circumvent the CD47-SIRPα interaction and phagocytosis inhibition, Alvey et al. [68] engineered human bone marrow derived macrophages to inhibit SIRPα (THP-1 SIRPα KD) and to express specific antibodies on anti-cancer cells (anti-MUC1, Cetuximab), so-called “A’PB macrophages”. The therapy was delivered in vivo by intravenous or intraperitoneal injections and tested in a human lung cancer model generated by subcutaneous injection of cancer cells (A549) in immunocompromised NSG mice. The study showed several interesting results. First, the engineered macrophages effectively reached the tumor microenvironment after systemic injection. Second, two to three days after the homing to the TME, the engineered bone marrow-derived macrophages were reprogrammed to become immunosuppressive TAMs. In addition, SIRPα was overexpressed in the reprogrammed TAMs. This could be one of the factors associated with the CD47-mediated immunoescape mechanism. Third, the treatment demonstrated efficacy in killing cancer cells in vivo. Of note, the maximum tumor regression (40%) was reached from day 10 to 14, and further continuous injections of macrophages did not show any therapeutic improvements. Moreover, although the investigators highlighted the safety of the treatment in vivo, they also pointed out the potential risk of off-target phagocytosis and development of autoimmunity associated with the treatment because of the CD47 ubiquitous expression.

### Myeloid cells engineered to release anti-cancer drugs

Systemic delivery of chemotherapeutic agents is a minimally invasive way of treatment but demonstrated limited efficacy in cancer patients. The main pitfalls associated with these treatments are the difficulties to penetrate the blood brain barrier, loss of active molecules due to hepatic and enzymatic degradation, rapid clearance by renal filtration, modest accumulation at the tumor site, off-target distribution, the development of side effects and toxicity [69]. Encapsulation of chemotherapeutic agents into nanoparticles and drug delivery in situ represent some of the strategies that have been developed to overcome these issues, which constitute the most critical challenges in pharmacology. One of the latest developments for inducible drug delivery systems in cancer treatment is the use of autologous cells, mainly leukocytes, as drug carriers [70, 71]. The system is designed to deliver in situ the chemotherapeutic agents and improve the killing of cancer cells. On this regard, a team of scientists recently setup an innovative therapeutic concept which combines “chemo, photo, and immunotherapy” for the treatment of primary and bone-metastatic breast cancer in combination with immunocheckpoint blockade [72]. This type of tumor is associated with low survival and no specific treatments are available. The novel therapy was based on engrafting tumor bearing immunocompetent mice with primary bone marrow-derived macrophages (iv injection) engineered to deliver nanoparticles containing oxaliplatin prodrug, a chemotherapeutic agent used against bone metastatic breast cancer cells. The activation of the cytotoxic agents was induced in vivo by exposure to near-infrared laser irradiation (NIR, chemo-photodynamic therapy). The therapy was tested in subcutaneous and intra-tibia breast cancer models in combination with anti-PD-L1 and demonstrated the tumor progression arrest and reduced bone-metastasis.

A similar therapeutic approach was evaluated for the treatment of colon carcinoma in vivo. The authors designed a sophisticated therapeutic concept, where a macrophage cell line (RAW 264.7) was engineered for the expression of the non-secreted form of TNFα and the induction of photothermal effects when the cells were irradiated with near-infrared radiation [73]. The cells were systemically injected in vivo and tracked to verify the homing to the TME. Following the injection of engineered macrophages, animals were irradiated at near-infrared frequencies at the tumor site. The radiation generated heat and triggered the photo thermolysis in the therapeutic myeloid cells, which, in turn, released the non-secreted form of TNFα, known for the cytotoxic effect on cancer cells. The authors demonstrated a positive effect on the survival of tumor bearing animals and a reduction of the toxicity associated with TNFα in vivo [74].

A different drug delivery system mediated by myeloid cells was designed to modify human macrophages by anchoring lipopolysaccharides on the plasma membrane (LMs). LMs were loaded with doxorubicin (DOX) [75] and tested in vivo in an orthoptic human lung cancer model. The treatment, systemically delivered, demonstrated tumor tropism but little intrapulmonary penetrance. The therapeutic protocol was started 7 days post-tumor implantation and iv injections of engineered macrophages were executed every 3 days, for 2 weeks. Animals treated with the LM-DOX macrophages showed a significant reduction of the tumor burden, reduction of pulmonary nodules and liver metastasis, and increased survival. Interestingly, LM-DOX macrophages induced the activation of TAMs to express TNFα in vivo, therefore enhancing the cytotoxic effect of the doxorubicin. These studies demonstrated that the use of macrophages as drug carriers for cancer treatment was effective and well tolerated. Therefore, myeloid cells could be used for the delivery of factors to the tumor site of various encapsulated chemotherapies and could be potentially used to develop novel therapeutic strategies that enhance current standard of care therapies.

## Conclusions

This article summarizes the most relevant investigations published so far on the use of engineered myeloid cells in cancer therapy (Fig. 1, Table 1).

The mechanisms of tumorigenesis and cancer progression are strictly dependent on the tridimensional architecture of the supportive tumor microenvironment. In addition, the direct interaction with cancer cells or with circulating factors released by cancer cells (cytokines, exosomes), can determine the switch of myeloid cells from pro-inflammatory to immunosuppressive phenotypes [76]. The immunosuppression mediated by myeloid cells contribute to the obstruction of trafficking and activation of cytotoxic T, NK, and DC cells, and the development of immunoescape mechanisms. Consequently, myeloid cells have been historically evaluated as a potential target for immunotherapies [10, 77, 78]. Based on the lessons learned on the crosstalk between myeloid cells and cancer cells, the pre-clinical development of novel therapies based on engineered myeloid cells for the treatment of cancer have been explored with success in the last two decades. Compared with recombinant and pro-inflammatory cytokines, or with CAR-T cells, the trafficking to the TME, target specificity, tolerance, and lower toxicity in vivo are among some notable advantages for the development of myeloid-based cellular therapies cells in cancer. Primary myeloid cells can be engineered to 1. release pro-inflammatory factors for the recruitment and activation of anti-cancer immune cells within the TME, 2. silence the expression of genes involved in immunoescape mechanisms, 3. potentiate phagocytosis 4. release anti-cancer molecules, or 5. deliver chemotherapies (Table 1, Fig. 1). Moreover, autologous myeloid cells can be modified to release a combination of different cytokines or chemotherapies. Considering the translation to the clinic and the testing of the therapeutic concept in clinical trials, we can conclude that the delivery can be performed systemically, the release of the compound can be tuned during treatment to minimize the toxicity and the treatment can be used in combination with other chemo/immune therapies. While promising, it is important to note that some studies have shown limitations of engineered myeloid cells in cancer, given myeloid cells recruited to the TME can be reprogrammed to become immunosuppressive and some of the tested therapies needed multiple treatments based on the limited cell survival in vivo (8–20 days). Moreover, systemically injected engineered cells showed off target distributions in different organs, thus affecting the therapeutic efficacy. In addition, some of the studies reached therapeutic significance only with intratumoral delivery, which can be problematic for the treatment of certain kinds of solid tumors including CNS tumors. Another aspect to consider is the lack of in vivo studies employing immunocompetent murine models. This is fundamental for testing cell-mediated immunotherapies in vivo. Also, the therapeutic approach was tested mostly on solid tumors, and there is a paucity of scientific investigations on hematological malignancies. The regulation of the cytokine release by myeloid cells is the key to minimize toxicity in vivo, but currently there are not enough translational studies in this space. Further, there is a need to expand the limited number of clinical trials with this approach (Table 2) to better evaluate toxicity and autoimmunity in addition to validating the therapeutic activity in cancer patients. Interestingly, the therapeutic properties of engineered macrophages have also been explored for the treatment of other diseases. Of note, translational studies on the use of engineered myeloid cells for the treatment of multiple sclerosis (MS) [79] and pulmonary infections [80] were recently published.

In conclusion, the use of engineered myeloid cells for the treatment of cancer is a new and promising therapeutic concept, but additional rigorous studies are necessary to validate their translational potential as a next-generation innate immunotherapy approach for patients with cancer.
